# Antioxidative Defense System, Hormones, and Metabolite Accumulation in Different Plant Parts of Two Contrasting Rice Cultivars as Influenced by Plant Growth Regulators Under Heat Stress

**DOI:** 10.3389/fpls.2022.911846

**Published:** 2022-05-27

**Authors:** Hassan S. Al-Zahrani, Hesham F. Alharby, Shah Fahad

**Affiliations:** ^1^Department of Biological Sciences, Faculty of Science, King Abdulaziz University, Jeddah, Saudi Arabia; ^2^Hainan Key Laboratory for Sustainable Utilization of Tropical Bioresource, College of Tropical Crops, Hainan University, Haikou, China; ^3^Department of Agronomy, The University of Haripur, Haripur, Pakistan

**Keywords:** antioxidants, cultivars, high night temperature, hormones, metabolites, plant growth regulators

## Abstract

We examined the metabolic, hormonal, enzymatic, and non-enzymatic responses of various plant components (leaf, root, and xylem sap) to plant growth regulators [methyl jasmonate (MeJA), ascorbic acid (Vc), brassinosteroids (Br), triazoles (Tr), alpha-tocopherol (Ve), and control] under heat stress [ambient temperature (AT), heat stress at night time (HNT), and heat stress at day (HDT)] in heat-sensitive (IR-64) and heat-tolerant (Huanghuazhan) rice cultivars under greenhouse conditions. Our results showed that heat stress altered the antioxidant activities and hormonal balance and rigorously reduced total soluble sugars, proteins, and proline, whereas increases were observed in H_2_O_2_ and Malondialdehyde (MDA) content accumulation in the plant xylem sap and leaves of both tested cultivars; however, the impact was more pronounced in IR-64. The superoxide dismutase (SOD), peroxidase (POD), catalase (CAT), ascorbate peroxidase (APX), glutathione reductase (GR), Glutathione (GSH), dehydroascorbate reductase (DHAR), and monodehydroascorbate reductase (MDHAR) activities were higher in Huanghuazhan than in IR-64 in response to temperature stress, when compared to AT. Additionally, heat stress increased abscisic acid (ABA) levels in both rice cultivars, especially in IR-64. The highest concentrations of hormones were recorded in the roots, followed by the leaves and xylem sap, in both cultivars. HDT and HNT stresses severely reduced the concentrations of all of the cytokinin types (except for iP9G and tZ9G) and IAA in the different plant parts of rice cultivars. Moreover, HNT was more detrimental for hormone and metabolite synthesis in both cultivars. The growth regulators (especially Vc + Br + Ve + MeJA) were comparatively more effective in minimizing the hostile impact of heat stress on most of the studied traits and should be applied to obtain the optimum yield of rice in subtropical and tropical areas under changing climatic conditions.

## Introduction

Heat stress is emerging as the main plant growth- and yield-limiting factor, especially in subtropical and tropical regions throughout the world. Global warming alters the optimal growth conditions for crops and thus adversely affects agricultural productivity. The Intergovernmental Panel on Climate Change (IPCC) has projected a 2–4°C increase in global average temperature by the end of the 21st century due to greenhouse gas emissions and anthropogenic and/or natural factors (Eitzinger et al., 2010; Smith and Olesen, 2010). Recently, global warming has resulted in crop exposure to higher temperatures and has altered crop yield potential. Therefore, it is necessary to develop specific cultivars that are resistant to high temperature (Moreno and Orellana, 2011). Plants can vary in their responses to high temperature depending upon the plant type, cultivar, extremity, and duration of heat stress; however, the correct identification of traits that induce heat stress is still unknown (Wahid et al., 2007; Fahad et al., 2014a,b, Fahad et al. (2015a,b), Fahad et al., 2016a,b,c,d, Fahad et al., 2017, 2018; Atif et al., 2021). High temperature alters protein and membrane stability, RNA types and cytoskeleton structures; additionally, it reduces enzymatic activity and produces metabolic imbalances (Ruelland and Zachowski, 2010); Ahuja et al. (2010) reported that under extremely high temperatures, plant cells may die within minutes and may cause cellular destruction.

Temperature and photoperiod have major roles in the development and growth of crops (Kropff et al., 1995; Fahad et al., 2016a, b,c,d, 2020, 2021a,b,c,d,e,f; Afzal et al., 2020, 2022; Ahmad et al., 2021; Ali et al., 2021; Haider et al., 2021; Javed et al., 2021). Rice requires 27–32°C for its optimal growth (Yin et al., 1996). Plants minimize oxidative damage via many natural defense systems under optimal growth conditions. For example, they maintain reactive oxygen species (ROS) at lower concentrations, either via the production of antioxidant enzymes such as peroxidase (POD), ascorbate peroxidase (APX), superoxide dismutase (SOD), catalase (CAT), and glutathione reductase (GR) (Fahad et al., 2017) or via the regeneration of oxidized antioxidants such as dehydroascorbate and glutathione (Markovska et al., 2009). The POD, CAT, superoxide, and APX enzymes play a significant role in protecting plants from high-temperature stress damage by scavenging ROS. When superoxide radicals are converted into H_2_O_2_ by SOD, they may be reduced to water or oxygen (either by APX or CAT) inside the cell (Howarth, 2005). These antioxidant enzymes have been reported to enhance their expression under biotic and abiotic stresses (Rizhsky et al., 2004; Sharma and Dubey, 2005).

Heat stress adversely affects the stability, contents, synthesis, and compartmentalization of hormones in plants (Maestri et al., 2002; Fahad et al., 2016a, b,c, d, 2020, 2021a,b,c,d,e,f; Mahmood et al., 2021). Abscisic acid (ABA), which is a signaling molecule, is involved in the activation of several physiological processes under stress conditions. Under stress conditions, plants enhance ABA production (Larkindale and Huang, 2004). In contrast, the production of cytokines such as isopentinyl adenosine (iPA), dihydrogen zeatin riboside (DHZR), zeatin (Z), and zeatin riboside (ZR) decreases under heat stress (Liu and Huang, 2005), which reduces plant reproduction, growth, and yield (McClung and Davis, 2010; Mittler and Blumwald, 2010; Hasanuzzaman et al., 2013) depending on the growth stage (Wahid et al., 2007), Farrell et al. (2006) have reported flowering and booting as being the most susceptible stages to heat stress in rice.

Rice production is dependent on rising temperatures. China [especially the Yangtze River Valley (YRV), which occupies 70% of the rice-producing area (in China) and is a main rice producer Dong et al., 2011; Fahad et al., 2016a,b,c,d], greatly contributes to food security. However, rice yield in the YRV declines due to heat stress, especially in the mid-season, wherein the temperature seldom increases above 35°C (Matsui, 2009), thus suggesting that the critical high temperature for rice yield reduction in the YRV is far lower than that in Australia (Tian et al., 2010), wherein heat exceeding 40°C occasionally reduces rice yield (Matsui et al., 2007).

Growth regulators, such as brassinosteroids (Br), alpha-tocopherol (Ve), methyl jasmonate (MeJA), ascorbic acid (Vc), and triazoles (Tr), that were used in our experiments are mainly associated with heat tolerance and plant protection against oxidative damage (Mohammed and Tarpley, 2011). Ascorbic acid is an antioxidant metabolite that plays a vital role in photosynthesis, cell division, gene regulation, the modulation of flowering time, senescence, and photoprotection (Hager and Holocher, 1994; Noctor and Foyer, 1998). It also induces drought, heat and cold stress tolerance in plants (Guo et al., 2005). It also decreases ROS production by slowing glutathione in the ascorbate glutathione cycle (Noctor et al., 2002). Similarly, vitamin E (a-tocopherol) is the most effective and strong antioxidant (Munne-Bosch and Alegre, 2002). Tolerant plants maintain high levels of tocopherol, whereas sensitive species reduce tocopherol production under stress, thus resulting in cell destruction and oxidative damage (Munné-Bosch and Alegre, 2003). As growth regulators, BRs have a significant role in promoting photomorphogenesis, cell elongation, seed germination and xylem differentiation (Krishna, 2003; Sasse, 2003; Koh et al., 2007). Additionally, BR induces heat, drought, cold, and salinity tolerance in plants via genetic modification in genes such as *RD29A, ERD10*, and *heat shock protein* (*hsp*) genes (Kagale et al., 2007). Similarly, MeJA is a cell regulator (Wasternack and Hause, 2002) that enhances the ability of plants to tolerate abiotic stresses (Brossa et al., 2011). According to Chen et al. (2011), MeJA-treated plants exhibit a modified protein profile, which improves plant physiological processes such as photosynthesis and carbohydrate anabolism (Percival and Noviss, 2008).

The application of growth regulators such as Vc, Ve, Br, MeJA, and Tr is an appropriate and economical approach for improving crop tolerance and performance under heat stress (Bavita et al., 2012; Zhu et al., 2013). However, their effectivity depends upon the type, species and plant growth stage. Therefore, the current study investigated the metabolic, hormonal, enzymatic, and non-enzymatic responses of different plant parts of two contrasting rice cultivars (sensitive and tolerant cultivars) under heat stresses imposed at day and night.

## Materials and Methods

### Experimental Procedure

Seeds of heat-sensitive (IR-64) and heat tolerant [Huanghuazhan (HHZ)] rice cultivars with the same architecture were placed in moist towels for 2 days and then planted (from 15 May 15 to 25 September) in seedling trays (at the rate of one seed per cell) under natural conditions. At 21 days after sowing, the seedlings were transplanted into plastic plots (12.6, 27.2, and 27.2 cm^3^ at lower inside, outside diameter and height, respectively) containing 12 kg of soil. The cultivar IR-64 was cultivated two weeks earlier than HHZ to match their heading times. A total of 10 g of compound NPK fertilizer was uniformly applied to all of the pots. Standard agronomic and cultural practices that were suitable for pot experiments were adopted throughout the duration of the experiment.

### Treatment Details

In this pot experiment, we examined the metabolic, hormonal, enzymatic and non-enzymatic responses of two contrasting rice cultivars [IR-64 (heat sensitive) and Huanghuazhan (heat tolerant)] to plant growth regulators (Vc, MeJA, Br, Ve, Tr, and control) under heat stress [high day (HDT), high night (HNT), and ambient temperature (AT)] under greenhouse conditions. The growth regulators Vc, Br, Ve, MeJA, and Tr were used at concentrations of 1.4, 4.0, 6.9, 1.0, and 0.55 mg L^–1^ in solution, respectively, in five different combinations, including (1) Vc + Br + Ve + MeJA, (2) Br + MeJA + Tr, (3) Vc + Ve, (4) MeJA alone, and (5) absolute control (AC). These combinations were used three times at 28, 35, and 42 days after germination prior to imposing the high temperature stresses. To impose heat stress, three growth chambers were adjusted at three different temperatures, including AT (28°C ± 2 for 24 h a day), HNT (32°C ± 2 from 7:00 pm to 7:00 am for 12 h), and HDT (35°C ± 2 from 7:00 am to 7:00 pm for 12 h). Plants were subjected to the abovementioned heat treatments from the booting stage until physiological maturity. The humidity was maintained at 75%, whereas the light was maintained at 1,000 μM m^–2^ s^–1^ inside the growth chamber throughout the experiment. The pots were regularly randomized at 15-day intervals inside the growth chamber to provide homogeneous environmental conditions.

### Xylem Sap Extraction

Plant xylem sap was extracted by using the procedures of Dodd et al. (2004) and Rahayu et al. (2005), with slight modifications. Therefore, the plant stem was cut 2 cm above the soil surface, washed and stored in a bottle with filter paper. Afterward, the sap was pumped into a silicon tube by applying root pressure for 3 h and maintained on ice for short intervals to avoid tube overflow. Finally, the sap was stored at −20°C for analysis.

### Observations

Total soluble sugars (TSS) in the xylem sap and leaves were measured via the phenol-sulfuric acid method (Dubois et al., 1956). For protein and proline determinations in xylem sap, we used the procedure of Bradford (1976), Gilmour et al. (2000), respectively. The Malondialdehyde (MDA) content in xylem sap that was collected from all of the treatment plants was measured by using the procedure of Hendry et al. (1993). The H_2_O_2_ concentration in sap was measured by using the procedure of Velikova et al. (2000). We adopted the Bai et al. (2009) method for measuring SOD, POD, and CAT activities, whereas APX activity was measured by using the Nakano and Asada (1981) procedure. Glutathione reductase (GR) was quantified by using the procedure of Foster and Hess (1980). Glutathione (GSH) and glutathione disulfide (GSSG) contents were measured by using the method of Smith (1985). Similarly, to measure ascorbate (ASC), we adopted the method used by Foyer et al. (1983). The changes between the total ascorbate and ascorbate contents were considered as dehydroascorbate (DHA). Dehydroascorbate reductase (DHAR) activity was examined by using the Hossain et al. (1984) procedure.

The ABA, IAA, and different types of cytokinin contents, including dihydrozeatin riboside (diHZR), dihydrozeatin (diZ), isopentenyl adenosine 5′-monophosphate (iPMP), isopentenyl adenine 9-glucoside (iP9G), isopentenyl adenosine (iPA), and isopentenyl adenine (iP), as well as trans-zeatin 9-glucoside (tZ9G), zeatin (Z), and zeatin riboside (ZR), in the xylem sap, were determined by using the protocols of Kettner and Doerffling (1995); Takei et al. (2001), and Walch-Liu et al. (2000), with slight changes as described by Lu et al. (2007).

### Statistical Analysis

The replicated (4) data were processed by using a factorial (2) complete randomized design (CRD) for analysis of variance (ANOVA), followed by the least significant difference (LSD) test for further comparisons among the means through the use of the statistical software Statistix 9.0.

## Results

### Metabolites and Reactive Oxygen Species

Total protein, proline, and soluble sugars (TSS) in leaves (Table 1) and xylem sap (Table 2) of both rice cultivars were considerably influenced by heat stress and PGR application (*p* ≤ 0.05). However, their interaction was non-significant, except for xylem sap and total proteins in cultivar IR-64 (Table 2). The cultivar IR-64 produced lower soluble sugar, total protein and proline contents in both plant parts than Huanghuazhan (Tables 1, 2). In both tested cultivars, the production of all of these metabolites in leaves was higher than that in xylem sap. Similarly, the TSS, protein and proline contents in both cultivars decreased with exposure to HDT and HNT (Tables 1, 2); however, the impact of HNT on these traits was more prominent than that of HDT. PGRs were helpful in minimizing the ill effects of heat stress on these features. Furthermore, higher TSS, protein and proline concentrations in leaves (Table 1) and xylem sap (Table 2) of both rice cultivars were recorded in plants treated with Vc + Br + Ve + MeJA, followed by Vc + Ve, Br + MeJA + Tr and MeJA, whereas the lowest values were observed under the control treatment, for both rice cultivars.

**TABLE 1:** Total soluble sugars (TSS), proteins, proline, H_2_O_2_, and MDA accumulation in the leaves of two rice cultivar as affected by plant growth regulators under heat stress at heading.

Temperature	PGR	TSS (g kg^–1^)		Total proteins (g kg^–1^)		Proline contents (g kg^–1^)		H_2_O_2_ (nmol g^–1^)		MDA (nmol g^–1^)	
		IR-64	HHZ	IR-64	HHZ	IR-64	HHZ	IR-64	HHZ	IR-64	HHZ
HNT	Vc + Br + MeJA Ve	14.88	30.13	6.22	12.53	1.16	1.52	77.35	74.50	74.13	57.50
	Br + MeJA + Tr	12.50	28.50	4.47	10.28	1.11	1.46	82.33	78.83	79.34	62.22
	Ve + Vc	13.50	29.50	5.22	11.40	1.14	1.49	79.33	75.33	76.91	60.00
	MeJA	11.25	27.50	3.72	9.40	1.09	1.4	85.17	81.00	82.12	64.58
	AC	10.13	26.88	3.10	8.78	1.06	1.4	87.83	83.17	84.72	66.67
HDT	Vc + Br + MeJA Ve	22.63	32.75	10.10	14.90	1.28	1.55	72.67	63.33	61.28	52.64
	Br + MeJA + Tr	19.50	31.63	8.22	13.15	1.22	1.51	77.67	68.67	66.67	57.08
	Ve + Vc	18.63	32.63	9.22	13.90	1.25	1.53	75.17	66.00	64.06	55.00
	MeJA	18.25	29.38	7.22	12.03	1.2	1.49	80.17	71.17	69.10	59.17
	AC	17.38	28.63	6.60	11.28	1.17	1.46	82.33	73.67	71.18	60.97
AT	Vc + Br + MeJA Ve	26.38	34.00	14.50	16.03	1.42	1.67	40.79	62.65	42.50	51.67
	Br + MeJA + Tr	24.63	31.75	13.38	15.15	1.37	1.6	42.89	65.15	44.85	54.31
	Ve + Vc	25.50	32.63	13.88	15.65	1.39	1.63	41.84	63.97	43.68	53.06
	MeJA	23.88	30.88	12.75	14.65	1.35	1.57	43.95	66.18	46.03	55.42
	AC	23.13	30.25	12.38	14.15	1.33	1.55	45.00	67.35	47.21	56.39
Temperature		**	**	**	**	**	*	**	**	**	**
PGR		*	*	**	**	*	*	*	**	**	*
Temperature × PGR		NS	NS	NS	NS	NS	NS	NS	NS	NS	NS
CV		9.91	8.42	8.44	9.84	6.27	7.52	6.62	7.13	9.91	7.34

PGR; CV; HHZ; IR-64; NS; ** and *; HDT; HNT, and AT stand for plant growth regulators; coefficient of variation; heat tolerant (HZZ); heat susceptible (IR-64); non-significant; significantly different at α = 0.01 and 0.05, high day; high night, and ambient temperature, correspondingly. Vc; MeJA; Ve; Tr; Br, and AC represent vitamin C, methyl jasmonates, vitamin E, triazoles; brassinosteroids, and control applied at the rate of 1.4, 1.8, 6.9, 0.55 4.0, and 0 mg L**^–^**^1^, respectively.

**TABLE 2:** Total soluble sugars (TSS), proteins, proline, H_2_O_2_, and MDA accumulation in xylem sap of two rice cultivar as affected by plant growth regulators under heat stress at heading.

Temperature	PGR	TSS (mg ml^–1^)		Total proteins (mg ml^–1^)		Proline contents (mg ml^–1^)		H_2_O_2_ (nmol ml^–1^)		MDA (nmol ml^–1^)	
		IR-64	HHZ	IR-64	HHZ	IR-64	HHZ	IR-64	HHZ	IR-64	HHZ
HNT	Vc + Br + MeJA Ve	6.25	12.33	4.95	8.70	0.74	1.16	60.67	59.29	61.25	44.56
	Br + MeJA + Tr	7.70	11.35	4.70	6.95	0.69	1.11	66.17	64.82	67.50	48.82
	Ve + Vc	8.75	12.18	4.08	7.83	0.72	1.13	63.33	62.37	64.58	46.76
	MeJA	6.88	10.75	3.45	6.08	0.66	1.09	67.50	67.68	68.13	50.59
	AC	5.38	10.13	3.41	5.33	0.63	1.06	70.00	70.18	70.83	52.35
HDT	Vc + Br + MeJA Ve	14.05	17.38	7.95	10.95	0.90	1.22	54.00	48.93	48.13	39.26
	Br + MeJA + Tr	11.83	16.13	5.83	8.95	0.85	1.18	59.17	54.11	53.96	43.68
	Ve + Vc	12.88	16.08	6.74	10.08	0.87	1.19	56.50	51.61	51.25	41.47
	MeJA	10.63	14.87	4.97	7.95	0.81	1.16	61.67	56.79	55.29	45.59
	AC	9.88	12.32	4.09	7.33	0.79	1.14	63.67	58.75	58.97	47.50
AT	Vc + Br + MeJA Ve	18.38	24.63	10.45	11.70	1.00	1.32	52.06	32.36	32.38	36.62
	Br + MeJA + Tr	16.50	23.38	9.20	10.45	0.94	1.27	54.50	34.72	34.67	38.97
	Ve + Vc	17.38	24.00	9.70	11.08	0.95	1.29	53.17	33.47	33.33	37.79
	MeJA	15.63	22.50	8.70	9.83	0.92	1.24	55.83	35.83	35.50	40.05
	AC	15.00	21.63	8.33	9.33	0.90	1.21	57.33	36.94	36.33	41.47
Temperature		**	**	**	**	**	**	**	**	**	**
PGR		**	**	**	**	*	*	**	**	**	**
Temperature × PGR		ns	ns	*	ns	ns	ns	ns	ns	ns	ns
CV		14.94	10.33	10.74	15.68	5.48	5.93	5.02	5.15	7.94	4.88

PGR; CV; HHZ; IR-64; NS; ** and *; HDT; HNT, and AT stand for plant growth regulators; coefficient of variation; heat tolerant (HZZ); heat susceptible (IR-64); non-significant; significantly different at α = 0.01 and 0.05, high day; high night, and ambient temperature, correspondingly. Vc; MeJA; Ve; Tr; Br, and AC represent vitamin C, methyl jasmonates, vitamin E, triazoles; brassinosteroids, and control applied at the rate of 1.4, 1.8, 6.9, 0.55 4.0, and 0 mg L**^–^**^1^, respectively.

Heat stress considerably enhanced MDA and H_2_O_2_ contents in both plant parts of the tested rice cultivars (Tables 1, 2). Nevertheless, such an increase was more pronounced under HNT than under HDT. When comparing the cultivars, IR-64 demonstrated higher MDA and H_2_O_2_ contents than Huanghuazhan under high temperature; however, MDA and H_2_O_2_ contents were higher in Huanghuazhan under AT. All of the PGRs demonstrated pronounced reductions in MDA and H_2_O_2_ contents in both plant parts, particularly under high-temperature stress. When averaged across different temperature treatments and two cultivars, minimum MDA and H_2_O_2_ contents were observed in plants supplemented with Vc + Ve + MeJA + Br, followed by Vc + Ve (Tables 1, 2).

### POD, SOD, CAT, and APX

**FIGURE 1:**
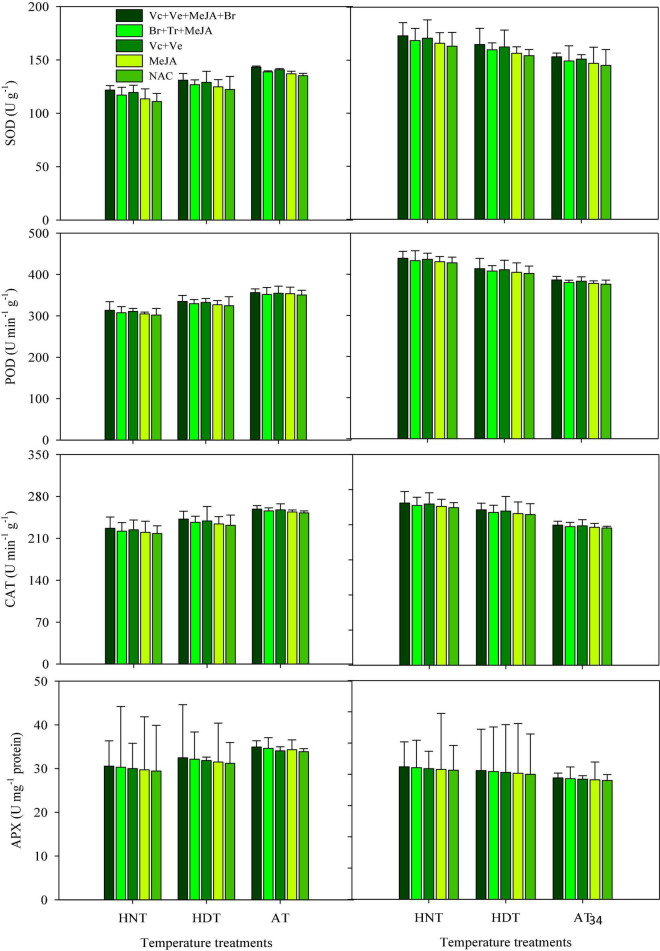
Catalase (CAT), ascorbate peroxidase (APX), superoxide dismutase (SOD), peroxidase (POD) accumulation in rice leaves as influenced by growth regulators under heat stress. HDT, HNT, and AT stand for high day, high night, and ambient temperature, respectively. The lines on bars represent LSD value for the interaction of PGRs and heat stress (heat stress × PGR) at α = 0.05.

**FIGURE 2:**
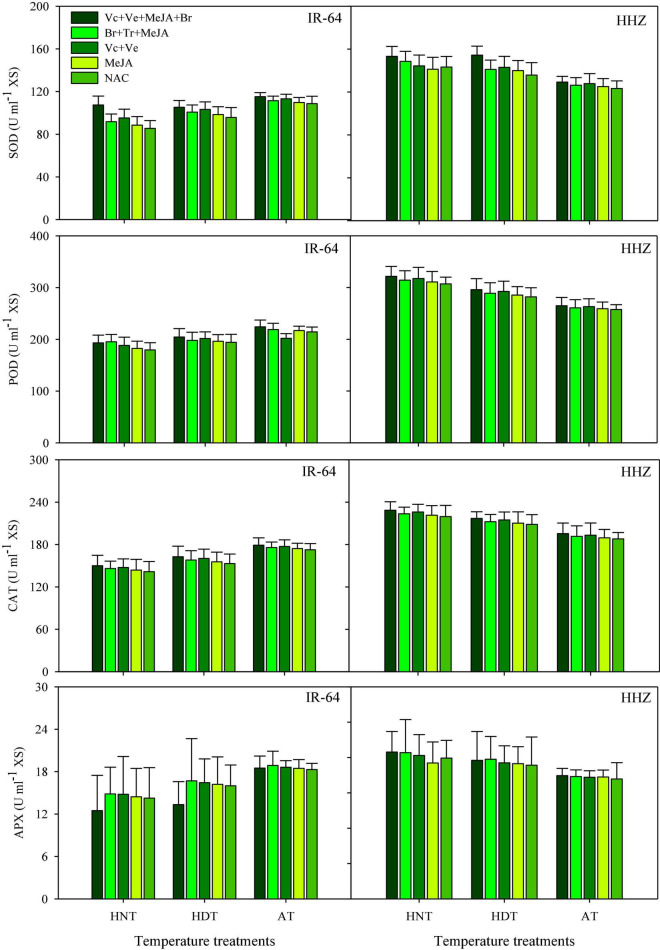
Catalase (CAT), ascorbate peroxidase (APX), superoxide dismutase (SOD), peroxidase (POD) accumulation in the xylem sap of rice as influenced by growth regulators under heat stress. HDT, HNT and AT stand for high day, high night, and ambient temperature, respectively. The lines on bars represent LSD value for the interaction of PGRs and heat stress (heat stress × PGR) at α = 0.05.

Catalase, SOD, POD, and APX activities in leaves (Figure 1) and xylem sap (Figure 2) at the heading stage significantly varied among the tested cultivars under heat stress, whereas their interaction effect was non-significant (Figures 1, 2). The effect of PGRs was only significant for SOD and POD activities in both plant parts. Irrespective of PGR application, the highest CAT, SOD, POD and APX activities in the leaves and xylem sap of susceptible cv. IR-64 were recorded under AT, which were then decreased upon exposure to HDT or HNT. In contrast, the maximum SOD, POD, APX, and CAT activities for tolerant cv. Huanghuazhan were observed under HNT. In Huanghuazhan, exposure to HDT or HNT resulted in higher CAT, SOD, POD, and APX activities in leaves and antioxidants, whereas a comparatively lower antioxidant activity was produced in IR-64 under heat stress, compared to AT. Generally, greater CAT, SOD, POD, and APX activities were observed in the leaves of both rice cultivars than in xylem sap. Similarly, PGR supplementation considerably improved SOD and POD activities in leaves (Figure 1) and xylem sap (Figure 2), thus showing their maximum rates in Vc + Ve + MeJA + Br-treated plants. None of the PGR treatments significantly enhanced CAT and APX activities in either rice cultivar. Moreover, the application of MeJA alone was least effective.

### GR, GSH, and GSSC

**FIGURE 3:**
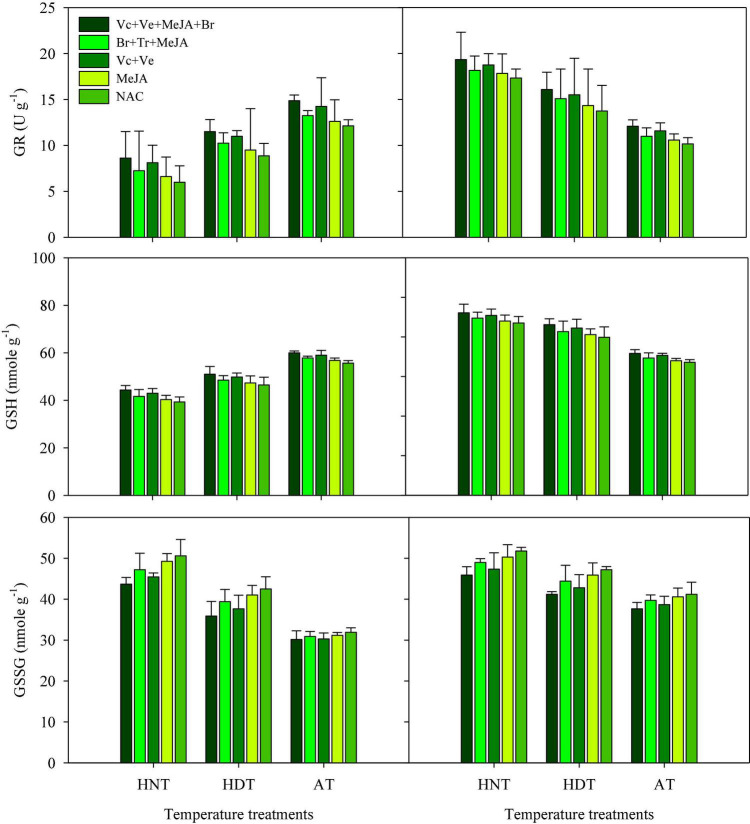
Glutathione (GSH), glutathione reductase (GR), and glutathione disulfide (GSSG) levels in rice leaves as affected by growth regulators under heat stress. HDT, HNT, and AT stand for high day, high night, and ambient temperature, respectively. The lines on bars represent LSD value for the interaction of PGRs and heat stress (heat stress × PGR) at α = 0.05.

**FIGURE 4:**
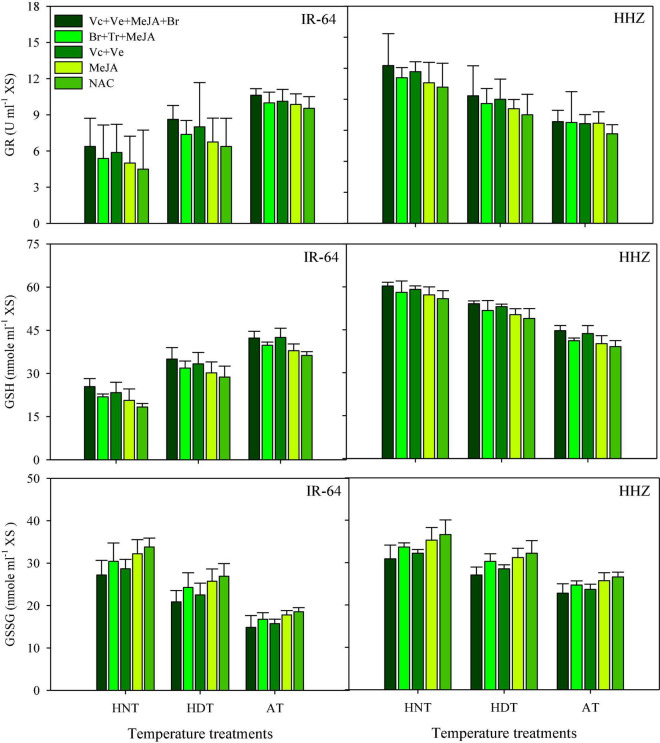
Glutathione (GSH), glutathione reductase (GR), and glutathione disulfide (GSSG) concentration in the xylem sap as affected by growth regulators under heat stress. HDT, HNT, and AT stand for high day, high night, and ambient temperature, respectively. The lines on bars represent LSD value for the interaction of PGRs and heat stress (heat stress × PGR) at α = 0.05.

High-temperature stress and exogenous application of PGRs resulted in significant alterations in the activities of GR, GSH, and GSSG in leaves (Figure 3) and xylem sap (Figure 4) for both rice cultivars (*p* ≤ 0.05). The responses of the two cultivars were also variable to high-temperature stress due to different sensitivity levels. In IR64, exposure to HDT or HNT resulted in significantly lower activities of GR and GSH in leaves (Figure 3), as well as in xylem sap (Figure 4), compared with AT. Nonetheless, the activity of both of these antioxidants was increased in both plant parts of Huanghuazhan upon exposure to high temperatures (Figures 3, 4). Furthermore, HDT and HNT stress resulted in higher GSSG activity in IR-64 and Huanghuazhan than in AT. When compared between plant parts, higher GR, GSH, and GSSC activities were noted in leaves than in xylem sap.

PGR application pronouncedly improved GR and GSH activities in leaves and xylem sap compared with the control (Figures 3, 4). However, significant reductions in GSSC activity were noted under the influence of exogenously applied PGRs. Mainly, the highest activities of GR and GSH were observed in both tested plant parts under the application of Vc + Ve + MeJA + Br. This PGR combination was also the most effective for reducing GSSC activity, irrespective of cultivar type. Moreover, Ve + Vc was the 2nd best PGR formulation after Vc + Ve + MeJA + Br. MeJA alone had less influence on these antioxidants than all of the other PGR combinations (Figures 3, 4).

### ASC, DHA, DHAR, and MDHAR

**FIGURE 5:**
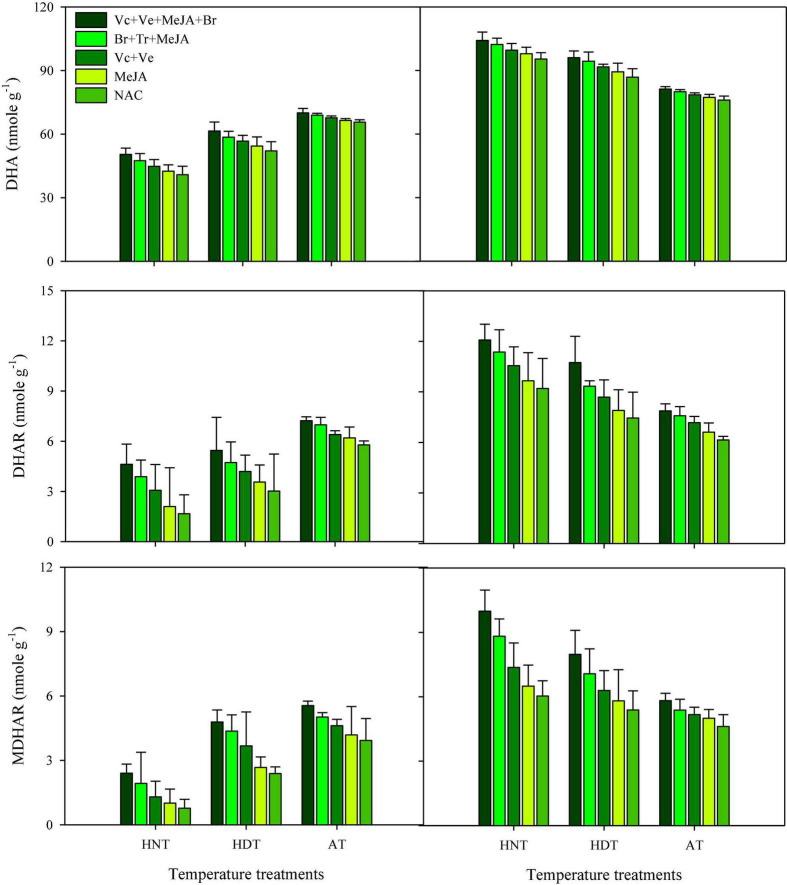
Monodehydroascorbate reductase (MDHAR), dehydroascorbate (DHA), and dehydroascorbate reductase (DHAR) concentration in rice leaves as affected by growth regulators under heat stress. HDT, HNT, and AT stand for high day, high night, and ambient temperature, respectively. The lines on bars represent LSD value for the interaction of PGRs and heat stress (heat stress × PGR) at α = 0.05.

**FIGURE 6:**
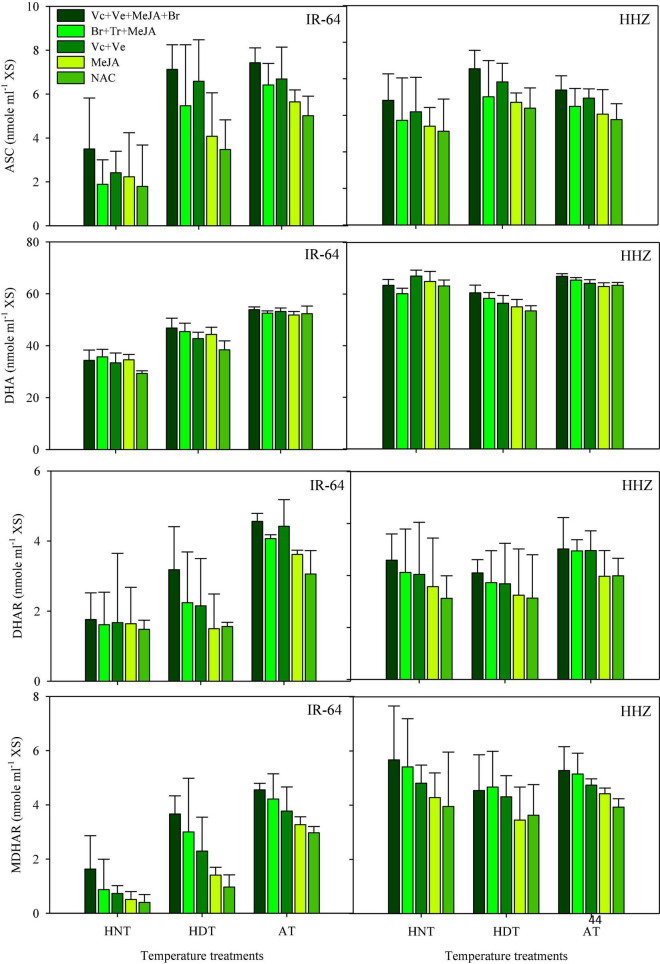
Monodehydroascorbate reductase (MDHAR), dehydroascorbate reductase (DHAR) and dehydroascorbate (DHA) activities in rice xylem sap in response to growth regulators under heat stress. HDT, HNT, and AT stand for high day, high night, and ambient temperature, respectively. The lines on bars represent LSD value for the interaction of PGRs and heat stress (heat stress × PGR) at α = 0.05.

Data concerning DHA, DHAR and MDHAR activities in the leaves and xylem sap are presented in Figures 5, 6, respectively. All of these antioxidants showed variable responses to different temperature and PGR treatments in both plant parts. The responses of heat-sensitive and heat-tolerant cultivars were also different in response to high day and night temperatures for these antioxidants. When averaged across different PGR combinations, the maximum DHA, DHAR, and MDHAR activities in the leaves, as well as the ASC, DHA, DHAR, and MDHAR activities in xylem sap of susceptible cv. IR-64, were recorded under AT (Figures 5, 6), which severely declined under HDT and HNT. In contrast, the maximum values for these antioxidants in tolerant cv. Huanghuazhan were noted under HNT, followed by HDT. In Huanghuazhan, exposure to high-temperature stress led to higher DHA, DHAR, and MDHAR activities; in IR-64, high temperature reduced the activities of these antioxidants, compared with AT.

The effect of various PGRs on DHA, DHAR, and MDHAR activities in both plant parts was significant in the tested cultivars. When compared with AC, PGR application considerably increased DHA, DHAR, and MDHAR activities in the analyzed plant parts of both cultivars (Figures 5, 6). Furthermore, the ASC activities in xylem sap were also increased under PGR supplementation in both varieties. In both plant parts, the maximum values of these antioxidants were observed when the Vc + Ve + MeJA + Br combination was applied. In leaves, this treatment was followed by the Br + Tr + MeJA application. For ASC and DHAR in xylem sap, Vc + Ve was the 2nd best treatment. Furthermore, MeJA alone was the less effective treatment in both plant parts, compared with all of the other PGR combinations.

### Hormone Contents

The cytokinin contents in different plant parts [viz., leaf (Table 3), root (Table 4), and xylem sap (Table 5)] of the two rice cultivars significantly varied in response to high-temperature stresses and PGR applications in the tested cultivars (*p* ≤ 0.05). Heat stress at night considerably reduced the concentrations of ZR, diHZR, diZ, Z, iP, iPA, and iPMP, whereas it increased in the tZ9G and iP9G concentrations in all of the plant parts. The lowest concentrations of ZR, diHZR, diZ, Z, iP, iPA, and iPMP were recorded for the plants under HNT. However, rice plants growing under AT showed the lowest values of tZ9G and iP9G. The cultivar IR-64 was comparatively more sensitive to heat stress than HZZ; therefore, it had lower concentrations of ZR, diHZR, diZ, Z, iP, iPA, and iPMP than Huanghuazhan. The tZ9G and iP9G responded differently than the rest of the cytokinins, and higher concentrations of tZ9G and iP9G were observed for IR-64 in all of the plant parts, compared with Huanghuazhan. The cytokinin contents also varied among the different plant parts of rice, and the highest concentrations were observed in the roots of both rice cultivars, followed by the leaves and xylem sap.

**TABLE 3:** Impact of plant growth regulators on hormones content in rice leaves under heat stress at heading stage.

Tem	PGR	TZ9G		ZR		DIHZR		DIZ		Z		IPMP		IP9G		IPA		IP	
		(ng g^–1^)																	
		IR-64	HHZ	IR-64	HHZ	IR-64	HHZ	IR-64	HHZ	IR-64	HHZ	IR-64	HHZ	IR-64	HHZ	IR-64	HHZ	IR-64	HHZ
HNT	Vc + Br + MeJA Ve	379.3	413.9	419.3	536.9	1.08	1.32	0.40	0.56	237.1	255.3	115.0	144.8	93.8	111.8	56.4	79.6	23.7	29.7
	Br + MeJA + Tr	387.2	421.1	416.0	530.8	1.03	1.31	0.39	0.55	234.8	252.3	105.7	138.6	98.0	115.2	53.5	77.6	20.7	28.0
	Ve + Vc	400.4	428.0	409.4	523.5	0.84	1.23	0.37	0.53	230.7	249.4	94.8	135.6	102.6	118.4	50.1	74.5	19.6	26.3
	MeJA	408.3	433.2	400.1	517.0	0.83	1.20	0.35	0.51	227.1	246.3	83.6	130.5	106.0	121.6	47.0	72.0	18.6	24.4
	AC	413.7	437.8	394.8	510.7	0.82	1.17	0.35	0.50	224.2	243.4	78.5	127.4	108.8	124.2	44.4	70.0	18.2	23.1
HDT	Vc + Br + MeJA Ve	372.1	407.7	436.4	547.5	1.21	1.38	0.48	0.58	242.0	259.6	118.8	160.3	83.6	107.8	64.5	85.3	25.9	31.9
	Br + MeJA + Tr	379.2	413.6	430.4	541.3	1.13	1.36	0.45	0.57	238.8	257.0	115.5	153.2	86.7	111.1	62.7	83.4	24.5	29.9
	Ve + Vc	388.5	421.5	423.8	531.4	1.08	1.34	0.42	0.56	236.3	253.7	105.7	147.3	92.5	114.9	59.2	80.0	22.1	28.2
	MeJA	395.5	427.2	415.3	525.2	0.98	1.33	0.39	0.53	232.6	250.5	98.4	138.2	96.4	117.4	56.6	76.8	21.0	26.5
	AC	403.2	432.0	410.0	519.0	0.96	1.32	0.38	0.52	230.0	248.6	92.4	135.1	99.9	119.6	54.1	75.0	20.0	25.2
AT	Vc + Br + MeJA Ve	363.1	401.1	453.1	553.2	1.31	1.54	0.54	0.69	251.0	263.3	134.4	163.0	77.4	104.2	69.2	87.2	30.2	33.8
	Br + MeJA + Tr	368.5	408.1	450.7	548.6	1.31	1.54	0.54	0.64	248.7	261.5	130.2	158.5	80.0	105.9	67.4	85.4	28.9	32.2
	Ve + Vc	377.5	414.7	447.8	545.4	1.27	1.53	0.48	0.60	246.3	258.9	127.3	153.8	82.1	108.0	65.9	83.4	27.2	30.3
	MeJA	383.9	419.5	443.8	540.5	1.22	1.52	0.46	0.58	243.1	256.4	124.1	148.3	85.2	111.4	64.2	81.6	25.4	28.9
	AC	387.1	423.7	439.9	533.9	1.21	1.49	0.43	0.56	241.0	255.0	117.1	141.2	87.6	114.1	61.9	79.5	23.8	27.3
Temperature		*	**	**	*	**	**	**	**	*	*	**	**	**	**	**	**	**	**
PGR		*	*	*	*	*	**	*	*	ns	ns	*	*	*	*	**	*	*	*
Temperature × PGR		ns	ns	ns	ns	ns	ns	ns	ns	ns	ns	ns	ns	ns	ns	ns	ns	ns	ns
CV		4.5	5.3	9.8	10.4	11.5	9.2	10.9	12.4	12.4	10.8	9.4	8.3	9.5	8.6	12.3	9.7	13.3	10.3

PGR; CV; HHZ; IR-64; NS; ** and *; IP9G; Z; TZ9G; TZ9G; DIZ; ZR; IPA; DIHZR; IP; IPMP; HDT; HNT and AT stand for plant growth regulators; coefficient of variation; heat tolerant (HZZ); heat susceptible (IR-64); non-significant; significantly different at α = 0.01 and 0.05, Isopentenyl adenine 9-glucoside, Zeatin, Trans-zeatin 9-glucoside, Dihydrozeatin, Zeatin riboside, Isopentinyl adenosine, Dihydrozeatin riboside, Isopentenyl adenine, Isopentenyl adenosine 5’-monophosphate, high day; high night, and ambient temperature, correspondingly. Vc; MeJA; Ve; Tr; Br, and AC represent vitamin C, methyl jasmonates, vitamin E, triazoles; brassinosteroids and control applied at the rate of 1.4, 1.8, 6.9, 0.55, 4.0, and 0 mg L**^–^**^1^, respectively.

**TABLE 4:** Impact of plant growth regulators on hormones content in rice root under heat stress at heading stage.

Tem	PGR	TZ9G		ZR		DIHZR		DIZ		Z		IPMP		IP9G		IPA			IP	
		(ng g^–1^)																		
		IR-64	HHZ	IR-64	HHZ	IR-64	HHZ	IR-64	HHZ	IR-64	HHZ	IR-64	HHZ	IR-64	HHZ		IR-64	HHZ	IR-64	HHZ
HNT	Vc + Br + MeJA Ve	421.3	458.4	574.8	696.6	1.38	1.73	0.62	0.85	348.4	371.5	115.2	156.8	68.0	90.5		41.4	62.9	10.2	16.7
	Br + MeJA + Tr	429.2	465.6	570.2	690.6	1.32	1.72	0.60	0.85	346.0	368.6	109.4	150.3	72.0	94.0		38.5	60.9	7.2	15.0
	Ve + Vc	442.4	472.5	565.9	683.2	1.13	1.65	0.58	0.82	341.9	365.7	101.1	145.2	76.3	97.1		35.1	57.8	5.3	13.3
	MeJA	450.3	477.7	557.4	676.8	1.12	1.61	0.56	0.80	338.3	362.6	94.6	141.7	79.8	100.3		32.0	55.3	3.4	11.4
	AC	455.7	482.3	552.8	670.4	1.11	1.58	0.56	0.79	335.4	359.6	90.5	138.7	75.5	103.0		29.4	53.2	2.3	10.1
HDT	Vc + Br + MeJA Ve	414.1	452.2	594.2	707.2	1.50	1.79	0.69	0.88	353.2	375.9	125.2	167.4	57.4	86.6		49.5	68.6	12.4	18.9
	Br + MeJA + Tr	421.2	458.1	588.1	701.1	1.43	1.77	0.66	0.87	350.1	373.2	120.4	161.4	61.3	89.8		47.7	66.6	11.0	16.9
	Ve + Vc	430.5	466.0	580.8	691.2	1.38	1.76	0.64	0.85	347.5	369.9	112.5	156.1	66.5	93.7		44.2	63.2	8.6	15.2
	MeJA	437.5	471.7	573.8	685.0	1.27	1.74	0.60	0.83	343.9	366.7	106.1	151.1	70.2	96.1		40.8	60.0	7.4	13.5
	AC	445.2	476.5	569.0	678.7	1.25	1.73	0.59	0.81	341.2	364.8	102.1	147.5	66.4	98.3		38.3	58.3	6.1	12.2
AT	Vc + Br + MeJA Ve	405.1	445.6	609.6	713.0	1.61	1.95	0.75	0.98	362.2	379.5	134.8	172.7	51.4	83.0		54.2	70.5	17.2	20.8
	Br + MeJA + Tr	410.5	452.6	607.2	708.3	1.60	1.95	0.75	0.94	360.0	377.8	131.9	168.1	53.3	84.7		52.4	68.6	15.9	19.2
	Ve + Vc	419.5	459.2	604.3	705.2	1.56	1.94	0.70	0.90	357.6	375.1	128.2	164.3	55.8	86.7		50.9	66.7	14.2	17.3
	MeJA	425.9	464.0	600.3	700.2	1.51	1.93	0.67	0.87	354.4	372.7	124.1	159.6	58.7	90.1		49.2	64.8	12.4	15.9
	AC	429.1	468.2	596.4	693.6	1.50	1.90	0.65	0.85	352.3	371.3	116.6	154.5	60.6	92.9		47.4	62.8	10.8	14.3
Temperature		**	**	**	*	**	**	*	*	*	**	**	**	**	**		**	**	**	*
PGR		*	*	*	*	*	*	*	*	*	*	*	**	**	*		*	*	*	*
Temperature × PGR		NS	NS	NS	NS	NS	NS	NS	NS	NS	NS	NS	NS	NS	NS		NS	NS	NS	NS
CV		9.3	8.8	10.3	11.3	7.9	6.7	9.4	13.3	8.4	10.3	10.5	14.5	9.5	10.7		9.5	9.9	11.8	12.1

PGR; CV; HHZ; IR-64; NS; ** and *; IP9G; Z; TZ9G; TZ9G; DIZ; ZR; IPA; DIHZR; IP; IPMP; HDT; HNT and AT stand for plant growth regulators; coefficient of variation; heat tolerant (HZZ); heat susceptible (IR-64); non-significant; significantly different at α = 0.01 and 0.05, Isopentenyl adenine 9-glucoside, Zeatin, Trans-zeatin 9-glucoside, Dihydrozeatin, Zeatin riboside, Isopentinyl adenosine, Dihydrozeatin riboside, Isopentenyl adenine, Isopentenyl adenosine 5’-monophosphate, high day; high night and ambient temperature, correspondingly. Vc; MeJA; Ve; Tr; Br, and AC represent vitamin C, methyl jasmonates, vitamin E, triazoles; brassinosteroids and control applied at the rate of 1.4, 1.8, 6.9, 0.55, 4.0, and 0 mg L**^–^**^1^, respectively.

**TABLE 5:** Impact of plant growth regulators on hormones content in xylem sap of rice under heat stress at heading stage.

Tem	PGR	TZ9G		ZR		DIHZR		DIZ		Z		IPMP		IP9G		IPA		IP	
		(pg ml^–1^ XS)																	
		IR-64	HHZ	IR-64	HHZ	IR-64	HHZ	IR-64	HHZ	IR-64	HHZ	IR-64	HHZ	IR-64	HHZ	IR-64	HHZ	IR-64	HHZ
HNT	Vc + Ve + MeJA + Br	664.3	717.9	522.5	724.5	24.4	37.4	13.0	23.2	218.5	297.2	43.9	58.1	36.8	52.0	59.5	93.0	21.6	29.6
	Br + Tr + MeJA	683.0	737.6	518.7	718.5	18.5	30.8	11.0	21.2	210.1	291.5	39.3	51.6	42.0	56.0	52.3	82.4	19.1	26.8
	VC + Ve	705.2	757.3	514.4	711.1	10.2	25.7	8.6	18.1	200.9	287.3	30.9	46.5	48.6	60.6	43.8	73.3	16.0	23.9
	MeJA	723.8	774.9	505.9	704.7	6.0	22.3	7.2	15.6	192.3	282.3	26.8	43.0	54.5	66.6	35.7	65.3	11.9	20.5
	AC	738.5	794.0	501.3	698.4	5.6	19.2	6.7	13.6	184.5	278.8	23.4	40.0	59.8	72.2	28.9	58.6	6.8	17.7
HDT	Vc + Ve + MeJA + Br	602.6	693.4	543.9	735.1	34.3	48.0	21.1	28.9	230.7	315.3	55.1	68.7	30.6	46.8	79.6	107.9	25.6	31.5
	Br + Tr + MeJA	615.4	707.9	537.7	729.0	29.5	42.0	19.3	27.0	223.5	309.2	50.2	62.7	34.7	51.1	72.3	99.5	22.2	29.1
	VC + Ve	634.0	725.5	530.3	719.1	21.7	36.6	15.8	23.6	219.0	302.9	42.4	57.4	39.8	56.7	65.6	88.3	19.3	26.7
	MeJA	650.8	740.2	522.8	712.9	15.3	31.6	12.4	20.4	212.7	293.4	36.0	52.4	44.2	62.6	54.7	78.1	15.8	23.7
	AC	672.4	755.5	517.5	706.6	11.2	28.0	9.9	18.6	205.0	286.5	32.0	48.8	49.9	66.1	44.7	68.6	12.7	21.6
AT	Vc + Ve + MeJA + Br	540.9	656.6	558.2	740.9	43.9	53.2	25.8	30.8	244.3	323.2	64.7	74.0	26.1	43.0	93.1	114.0	34.3	34.4
	Br + Tr + MeJA	552.3	666.1	555.7	736.2	41.1	48.7	24.0	29.0	237.5	317.2	61.8	69.4	28.8	47.4	84.5	107.5	30.4	32.6
	VC + Ve	565.0	682.2	552.8	733.1	37.4	44.8	22.5	27.0	232.2	310.8	58.1	65.6	31.1	51.5	77.2	100.3	27.2	30.7
	MeJA	580.6	694.0	548.8	728.1	33.2	40.1	20.8	25.2	229.9	307.3	54.0	60.8	35.7	55.1	70.1	89.9	22.2	28.6
	AC	599.3	709.2	544.9	721.5	25.7	35.0	19.0	23.1	229.3	303.5	46.5	55.8	40.6	59.6	62.0	83.1	17.8	26.2
Temperature		**	**	NS	NS	**	**	**	**	*	**	**	**	**	**	**	**	**	**
PGR		*	*	NS	NS	**	**	**	**	NS	NS	**	**	**	*	**	**	**	**
Temperature × PGR		NS	NS	NS	NS	NS	NS	NS	NS	NS	NS	NS	NS	NS	NS	NS	NS	NS	NS
CV		5.6	4.9	15.9	11.2	17.1	8.8	16.2	14.1	13.4	7.3	10.5	7.1	14.3	13.8	15.9	8.1	16.1	11.4

PGR; CV; HHZ; IR-64; NS; ** and *; IP9G; Z; TZ9G; TZ9G; DIZ; ZR; IPA; DIHZR; IP; IPMP; HDT; HNT and AT stand for plant growth regulators; coefficient of variation; heat tolerant (HZZ); heat susceptible (IR-64); non-significant; significantly different at α = 0.01 and 0.05, Isopentenyl adenine 9-glucoside, Zeatin, Trans-zeatin 9-glucoside, Dihydrozeatin, Zeatin riboside, Isopentinyl adenosine, Dihydrozeatin riboside, Isopentenyl adenine, Isopentenyl adenosine 5’-monophosphate, high day; high night and ambient temperature, correspondingly. Vc; MeJA; Ve; Tr; Br, and AC represent vitamin C, methyl jasmonates, vitamin E, triazoles; brassinosteroids and control applied at the rate of 1.4, 1.8, 6.9, 0.55, 4.0, and 0 mg L**^–^**^1^, respectively.

**FIGURE 7:**
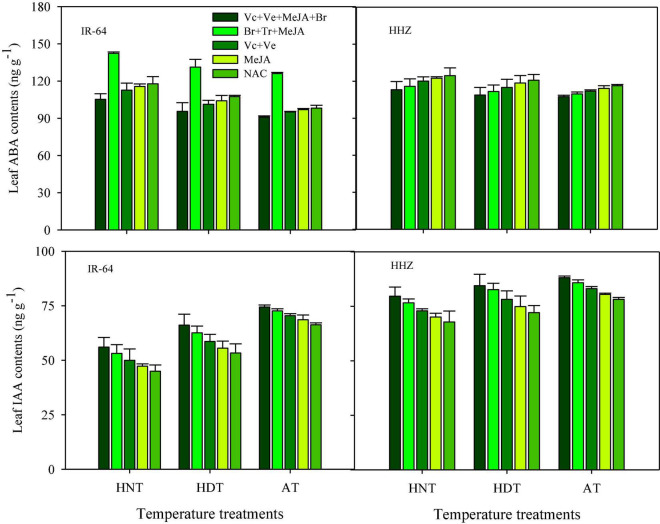
Impact of PGRs on the concentration of abscisic acid (ABA) and indole-acetic acid (IAA) in rice leaves.

**FIGURE 8:**
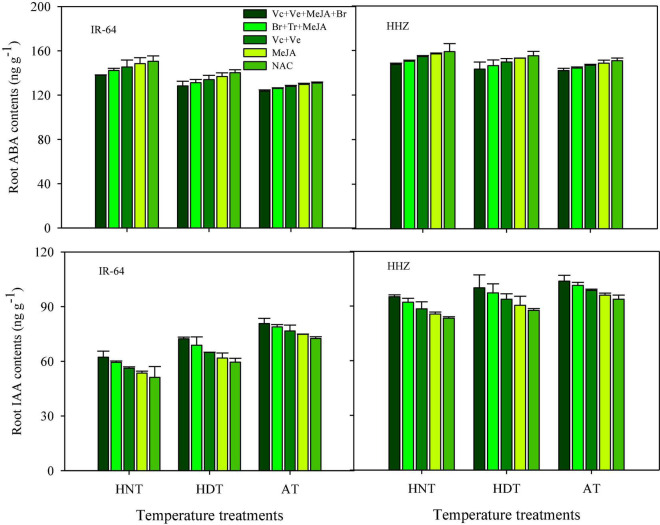
Impact of PGRs on the concentration of abscisic acid (ABA) and indole-acetic acid (IAA) in rice roots.

**FIGURE 9:**
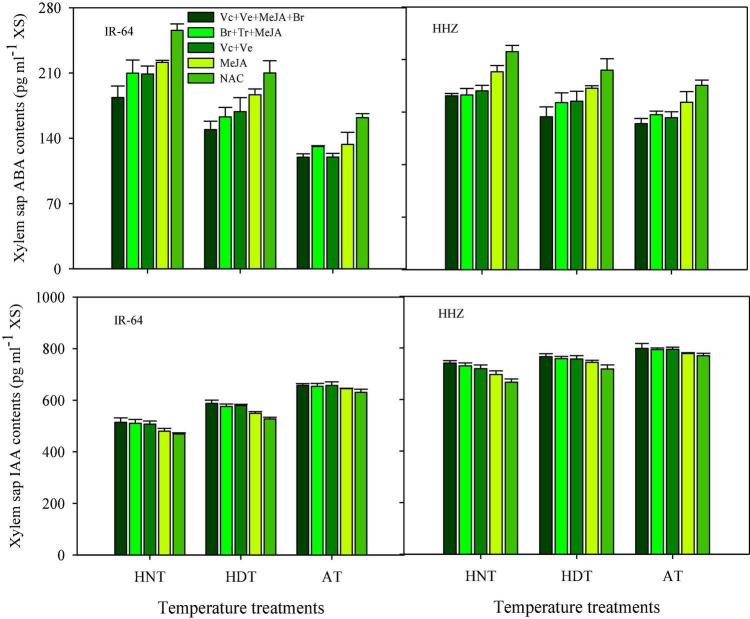
Impact of PGRs on the concentration of abscisic acid (ABA) and indole-acetic acid (IAA) in rice xylem sap.

Significant variations in the responses of ABA and IAA contents were also observed under the influence of HDT and HNT in leaves (Figure 7), roots (Figure 8), and xylem sap (Figure 9) of both rice cultivars. The ABA contents in all of the plant parts of the tested rice cultivars were significantly increased under heat stress. Nevertheless, HNT demonstrated more increments in ABA concentrations. Moreover, the response of IAA was the inverse of that of ABA at high temperatures. Both HDT and HNT significantly reduced the IAA contents in leaves, roots and xylem sap of both rice cultivars. Furthermore, the heat-susceptible cv. IR-64 recorded higher ABA contents and lower IAA contents under high temperature in all of the plant parts, compared with the tolerant cv. Huanghuazhan. When compared among the different plant parts, the highest ABA and IAA contents were recorded in roots, whereas the lowest values for these hormones were observed in the xylem sap of both cultivars.

The effects of PGRs were also significant on hormone concentrations in the different plant parts of rice cultivars, excluding Z and ZR in the leaves and xylem sap. However, their interaction was non-significant for all of the hormones. When compared with AC, the highest increases in ZR, diHZR, diZ, Z, iP, iPA, iPMP, and IAA concentrations were observed in different plant parts of both tested cultivars when Vc + Ve + MeJA + Br was exogenously applied. Moreover, this treatment demonstrated the lowest values for tZ9G, iP9G, and ABA contents in leaves, roots and xylem sap, and it was the most effective PGR combination under high-temperature stress.

## Discussion

Global warming is becoming a major challenge to food security throughout the world, as high temperatures diminish the performance of rice crops. Plant mechanisms to adapt/tolerate heat stress, such as antioxidative defense systems, metabolite synthesis and hormonal metabolism, have gained much attention, as they are mainly involved in protecting plants from heat injuries.

In the present study, we studied the influence of high-temperature stress on enzymatic and non-enzymatic antioxidants, metabolite synthesis, ROS generation and hormonal metabolism in different plant parts of two contrasting cultivars. Moreover, we investigated whether various PGRs applied in different combinations could induce heat tolerance in rice. HNT stress greatly reduced the concentrations of TSS, protein and free proline in both xylem sap and leaves, which has also been reported by Jain et al. (2007) and Bita and Gerats (2013). PGR supplementation was helpful in increasing metabolite synthesis in both rice cultivars. We observed higher metabolite synthesis in tolerant cv. Huanghuazhan compared with that in susceptible cv. IR-64. During investigations in sweet pepper, Saha et al. (2010) detected higher proline accumulation in heat-tolerant cultivars under heat stress than in heat-sensitive cultivars, which was further attributed to the higher and lower activities of proline biosynthesis and oxidizing enzymes, respectively.

In the present study, high-temperature stress (HDT and HNT) significantly increased lipid peroxidation, as well as H_2_O_2_ contents, in both tested rice cultivars. The production of ROS was lower in Huanghuazhan than in IR-64 under HDT and HNT, thus suggesting that Huanghuazhan cv. has an efficient radical scavenging system. Furthermore, this can also be related to higher protection against oxidative damage in Huanghuazhan via rapid scavenging or the removal of ROS. According to numerous researchers (Fahad et al., 2018, 2020, 2021a, 2021b, 2021c, 2021d, 2021e, 2021f), a higher accumulation of MDA is the most common cause of oxidative damage under high-temperature stress because high temperature induces ROS production, thus enhancing oxidative stress. We observed that MDA and H_2_O_2_ increased with heat stress compared to AT and showed a severe response under HNT treatments.

Tolerant plants have a well-established antioxidative defense system to counteract ROS-induced damage. These antioxidants are also involved in maintaining plant redox potential in plants. In the present study, we detected substantial variations in antioxidant activities in the tested plant parts of both studied cultivars due to heat stress. The cultivar Huanghuazhan exhibited higher GR, GSH, SODAPX, ASC, DHAR, POD, CAT, and MDHAR activities in the tested plant organs, whereas IR-64 exhibited lower antioxidant activity under heat stress. The higher tolerance in Huanghuazhan could be related to higher antioxidant activity even under stress conditions, whereas IR-64 is sensitive due to poor antioxidation. Moreover, greater antioxidant activity in the xylem sap and leaves of Huanghuazhan demonstrated that ROS production in Huanghuazhan was lower than that in IR-64, or that Huanghuazhan plants maintained higher antioxidant activities during HNT and HDT stress for cleansing ROS. Several studies have further confirmed the elevated POD and CAT in different crops under heat stress (Almeselmani et al., 2006).

High-temperature stress (particularly HNT) altered hormone production in leaves, roots and xylem sap in both tested cultivars. Reductions in iP, iPA, iP9G, Z, diZ, diHZR, ZR, and IAA and improvements in iPMP, tZ9G, and ABA were observed under stress. Yakushikina and Tarasov (1982), Fahad et al. (2017), have reported that endogenous levels of IAA and cytokinins were decreased and ABA concentration was augmented under heat stress. The level of CKTs is of prime importance for plant thermotolerance (Liu et al., 2002). Our study demonstrated increased production of ABA under heat stress. ABA has a significant role in stress sensing and signaling for plant protective measures (Larkindale and Huang, 2004). Moreover, ABA is a significant component of thermotolerance under field conditions, thus signifying its role in different pathways essential for thermotolerance in plants (Maestri et al., 2002). However, the responses of cytokinins and IAA to heat stress are in contrast to those of ABA. In bentgrass, the levels of different cytokinin types, such as ZR, Z, DHZR, and iPA, showed a significant decline in roots and shoots due to less dry matter production (Liu and Huang, 2005). Similarly, cytokinins regulate many important physiological and biochemical processes in plants (Fahad et al., 2015b). Specifically, they are involved in plant senescence (Yang et al., 2002), but inadequate evidence is available about the involvement of cytokinins in inducing heat stress in rice (Zhang et al., 2010). We observed increased glucoside cytokinins and a decrease in free bases and ribosides in all of the plant parts of rice upon the imposition of heat stress. Cytokinins have been used as plant tolerance indicators to abiotic stresses (Székács et al., 2000). Furthermore, wheat (Farkhutdinov et al., 1997), tomato and tobacco lines (Székács et al., 2000), as well as pea (Atanassova et al., 1996) plants, with higher cytokinin contents exhibited more tolerance to several stress factors.

In the present study, considerable variations among plant parts were recorded regarding cytokinin contents, and the highest concentrations were observed in roots, followed by leaves and xylem sap. Wagner and Beck (1993) observed higher cytokinins in roots than in the leaves and stems of *Urtica dioica* under heat stress. Additionally, they observed higher cytokinins in the roots than in stems and leaves. In addition, they also found cytokinins in the phloem sap of several plants (Wagner and Beck, 1993). This could be due to its higher supply by the roots to the shoots and leaves. Farkhutdinov et al. (1997) have reported decreased cytokinin accumulation in wheat seedlings under heat stress.

We observed that PGR application improved metabolite synthesis, antioxidant activities, IAA and cytokinin contents while reducing ABA levels and ROS production in different plant parts of both rice cultivars. Our findings are consistent with many researchers (Mohammed and Tarpley, 2009) who observed improved antioxidant and metabolite synthesis and reduced ROS production under PGR supplementation. Such improvements were due to enhanced antioxidant enzyme activities (Fahad et al., 2017). Mohammed and Tarpley (2009) observed a significant increase in antioxidant levels in response to PGRs such as vitamin E, salicylic acid and glycine betaine, thus avoiding membrane oxidative damage in plants. We observed a prominent positive effect of Vc + MeJA + Ve + Br in inducing heat tolerance in rice. The sole application of MeJA (Clarke et al., 2009), Vitamin C (Shah et al., 2001), Vitamin E (Munne-Bosch and Alegre, 2002), and Br (Zhang et al., 2007; Fahad et al., 2014a,2016a,2016c) in combating abiotic stresses has been previously reported. Our findings signify the role of PGRs as ROS scavengers, thus enhancing plant stability under stress and improving rice performance under heat stress. Antioxidant accumulation prevents enzyme degradation by ROS and promotes membrane stability. Furthermore, PGR application enhances the ability of plants to produce cytokinins and metabolites, thus improving plant tolerance to heat stress.

## Conclusion

Heat stress, especially high nighttime temperature, significantly reduced metabolite and hormone production, altered antioxidant activities and enhanced the production of ROS in different plant parts in both rice cultivars. More severe reductions in metabolites, IAA and different cytokinins (except iP9G and tZ9G) were observed in susceptible cv IR 64 compared with Huanghuazhan. Additionally, HDT and HNT increased non-enzymatic and enzymatic antioxidant activities in Huanghuazhan cultivars but reduced the activities of these antioxidants in IR-64 compared with AT. Furthermore, heat stress enhanced ABA accumulation in rice, especially in IR-64. The highest concentrations of hormones were recorded in the roots, followed by leaves and xylem sap, in both cultivars. Furthermore, PGR supplementation, especially Vc + Ve + MeJA + Br, significantly alleviated heat stress-related adversities in both susceptible and tolerant rice cultivars. Therefore, the application of PGRs (Vc + Ve + MeJA + Br) is recommended for obtaining the optimum yield of rice under changing climatic conditions.

## Data Availability Statement

The original contributions presented in the study are included in the article/supplementary material, further inquiries can be directed to the corresponding author.

## Author Contributions

HA-Z, HA, and SF: conceptualization. SF and HA: methodology, formal analysis, and supervision. HA: writing—original draft preparation. SF: writing—review and editing. HA-Z and HA: funding acquisition. All authors contributed to the article and approved the submitted version.

## Conflict of Interest

The authors declare that the research was conducted in the absence of any commercial or financial relationships that could be construed as a potential conflict of interest.

## Publisher’s Note

All claims expressed in this article are solely those of the authors and do not necessarily represent those of their affiliated organizations, or those of the publisher, the editors and the reviewers. Any product that may be evaluated in this article, or claim that may be made by its manufacturer, is not guaranteed or endorsed by the publisher.
